# Association of depressive symptoms with Geriatric Locomotive Function Scale score in community-dwelling older adults living in the state of emergency

**DOI:** 10.1186/s12877-023-04077-9

**Published:** 2023-05-31

**Authors:** Masakazu Imaoka, Misa Nakamura, Fumie Tasaki, Takao Inoue, Junya Orui, Ryota Imai, Mitsumasa Hida, Hidetoshi Nakao, Masatoshi Takeda

**Affiliations:** 1 Osaka Kawasaki Rehabilitation University, Osaka, Japan; 2 Josai International University, Chiba, Japan

**Keywords:** COVID-19, Depression, Geriatric, Pandemic, State of emergency

## Abstract

**Background:**

Under the state of emergency, it has been reported that the amount of physical activity among community-dwelling older adults has decreased significantly due to refraining from going out, and there are strong concerns about the Geriatric Locomotive Function Scale and deterioration of mental health. Therefore, this study aimed to investigate whether the depressive state before the coronavirus disease 2019 (COVID-19) pandemic affected the 25-Geriatric Locomotive (GLFS) score during the COVID-19 pandemic among community-dwelling older adults.

**Methods:**

The participants were 194 community-dwelling older adults (45 men, 149 women) with an average age of 75.5 ± 5.5 years who responded to a self-administered survey conducted three times (preliminary, second, and third) from before the 2018 COVID-19 pandemic to March 2021. Individuals with a score of ≥ 10 on the Geriatric Depression Scale 15 (GDS 15) were excluded. The survey items included the 25-question Geriatric Locomotive Function Scale (GLFS25), GDS 15, and other basic attributes. Those with scores of 5 to 9 on the GDS 15 and those with scores of 0 to 4 were assigned to the depressive symptoms (DS) group and the non-DS group, respectively. Statistical analysis was performed using two-way analysis of variance. The Mann–Whitney U test was used for comparisons between the groups.

**Results:**

In total, 187 patients were included in the analysis, excluding 7 patients. GLFS 25 showed a significant increase in scores at the second and third time points compared with baseline, and a main effect was confirmed in both groups, with no interaction effect. The second time, the score was 10.0 ± 8.5 and 13.7 ± 10.5 in the non-DS and DS groups, respectively. The third time, the non-DS and DS groups scored 10.8 ± 10.5 and 14.9 ± 10.1 points, respectively, indicating a significant difference.

**Conclusions:**

Our results revealed that the increase in the GLFS 25 score in community-dwelling older adults during the COVID-19 pandemic was related to their DS during normal times before the pandemic. Evaluating such individuals and providing social support may effectively reduce the deterioration of the GLFS 25 score.

## Background

The general term for the disease caused by the severe acute respiratory syndrome coronavirus 2 [1] is coronavirus disease 2019 (COVID-19), commonly known as the new coronavirus, which has become a global pandemic. In Japan, as of January 2023, 31,597,810 people have been infected, and the number of deaths has increased to 63320 [2]. Thus, people are still encouraged to wear masks in public transportation and public facilities [3]; compared with Europe and the United States, the infection control behavior of society as a whole has been continuing for a long time. The COVID-19 case fatality rate has decreased from 8.5% in February 2020 to 0.27% in August 2022, with an estimated 2.5-year relative risk reduction of 96.8% (95% confidence interval, 95.6–97.6; *p* < 0.001) [4]. Due to the high fatality rate, a state of emergency was initially declared for the first time in Japan, calling on the public to refrain from going out unnecessarily and to avoid close contact and crowds. This declaration markedly changed the behavior and lifestyle of the people [5–7]. A problem concerning this lifestyle is that minimizing going out will reduce the amount of activity of older adults and increase their risk of ill health. In particular, gatherings and lessons have been canceled in the community, and a state has been reached where hobbies and activities for purposes in life cannot be carried out. Yamada et al. [8] reported that as of April 2020, the amount of physical activity among community-dwelling older people had decreased by an average of 26.5% compared with normal times. Tison et al. [9] reported that the average number of steps taken worldwide decreased by 27.3%. Notably, the amount of physical activity decreases during the spread of infection but returns to the original level when the infection subsides; it is easier for people with high socioeconomic status and social participation levels before the pandemic to maintain the amount of physical activity [10]. A psychological research report stated that no psychosocial deterioration was confirmed in Japan as of August 2020 [11]. In contrast, the mental health of community residents deteriorated owing to anxiety, fear of infection, and insomnia [12]. It has been pointed out that not only mental health declines due to decreased opportunities for social participation and increased social frailty but also physical function deterioration are strongly affected [13]. In general, prevention is important because depression and depressive symptoms are more likely to develop in old age [14]. Moreover, having depressive symptoms is associated with a future decline in physical function [15]. At the same time, physical frailty is known to be a risk factor for future depressive symptoms and is known to influence each other [16, 17].

However, the impact of the pre-pandemic psychophysiological factors remains unclear. Previous reports have reported worsening depression due to loneliness in a pandemic [18]. Whether pre-pandemic depression has an impact is not particularly clear.

In particular, the depression rate among community-dwelling older people is as high as 30.3% [19], and individuals with depression and depressive tendencies are at risk of future frailty, sarcopenia, and the need for long-term care [20]. In addition, physical inactivity [21] against the background of depression is closely related to a sedentary lifestyle [22]; therefore, careful observation is important. In particular, infectious diseases, natural disasters, and accidents have made it impossible to go out and participate in society, as usual, affecting mental health. Moreover, mental health may affect physical and motor functions. Clarifying this may increase the importance of quantitative evaluation of depressive symptoms (DS).

Therefore, this study aimed to investigate whether DS during normal times would affect physical function during the 2020 and 2021 pandemics by including participants in a health checkup project targeting community-dwelling older people. Thus, we conducted a longitudinal survey of the changes in 25-Geriatric Locomotive Function Scale scores over time.

## Methods

### Participants

**Fig. 1:**
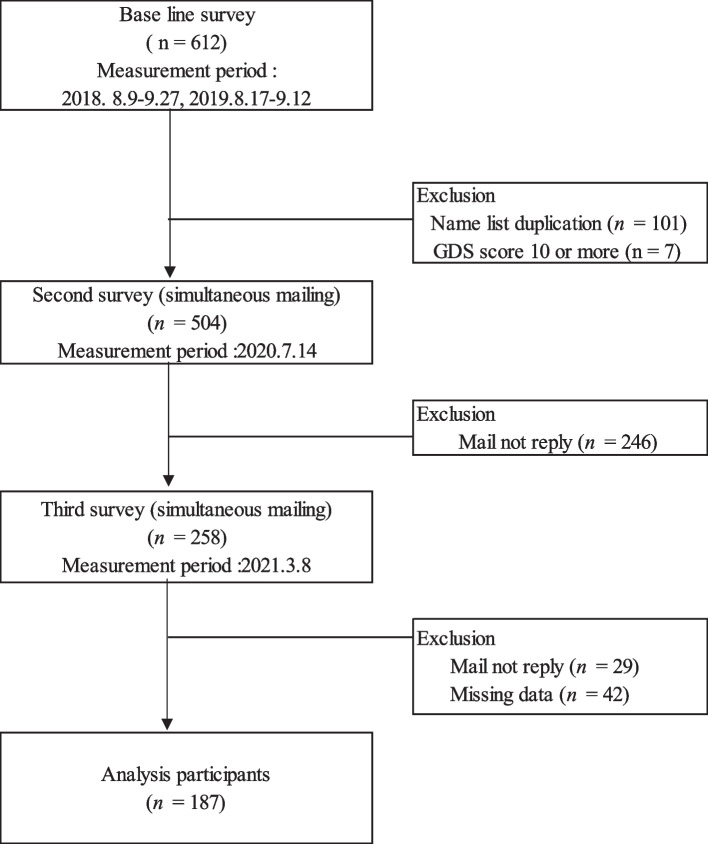
Study flow diagram

The participants were 504 community-dwelling older people aged ≥ 65 who participated in a health checkup project implemented as a dementia prevention project based on a comprehensive partnership agreement between the university and local governments (Fig. 1). Recruitment Methods For July 2018 and July 2029, flyers were placed at the city hall and community centers. During the same time, the city's public relations magazine was published to inform citizens of the recruitment. Exclusion criteria were as follows: those aged < 64 years, those with a score of ≥ 10 on the GDS 15, and those with possible depression. Kaizuka City in Osaka Prefecture, where the participants reside, is a small city with a population of 86,000. The city has an area facing the seaside and a Mountainous region with mountainous terrain. The city has a super-aged society with an aging population rate of 26.7%, and the percentage of the total population aged > 75 years is 14.0% (as of 2020).

The purpose of this study was explained to the participants in writing, and their consent was obtained. This study was approved by the research ethics review committee of Osaka Kawasaki Rehabilitation University (approval number: OKRU20-A013).

### Method

The survey was conducted using a two-way mail method via a self-report questionnaire. Approximately 2 months after the first state of emergency declaration was lifted, the mail was sent all at once on July 14, 2020, and on August 14 and March 10, 2021. Data that arrived on or before April 10 were included in the analysis. The survey items included GLFS- 25 [23, 24], grip strength [25], walking speed [26], extremity skeletal muscle mass index [27], depression [28], GLFS at the baseline health checkup in 2018 and 2019, general cognitive function [29], number of medications taken, and basic attributes. The 2020 and 2021 mail-in surveys determined GLFS-25 and assessed depression. Missing values in the mailed questionnaire were imputed using the multiple imputation method [30], and data were entered. Multiple imputation was performed on data with missing GLMS subitems in the 2020 and 2021 survey mailings; five data sets were created with GLMS scores, sex, age, and GDS-15 scores as auxiliary variables [31].

The GLFS 25 is a diagnostic tool for checking locomotor dysfunction and consists of 25 questions on pain, indoor movement, physical movement, activity participation, and anxiety. It is evaluated on a 5-point scale, with a total score ranging from 0 (no disability) to 100 (most severe disability), and determines the risk of requiring long-term care in the future. In this study, we recorded changes in the total score. Simultaneously, as GLFS is classified into healthy, GLFS1, GLFS 2, and GLFS 3 based on the cut-off, we also investigated changes in this classification. From the total score of GLFS 25, the cut-off value for Robust was 6 or less, GLFS 1 was 7 to 16 points, GLFS 2 was 16 to 24 points, and GLFS 3 was 24 points or more.

Grip strength was measured using a grip strength meter (Takei Kiki Kogyo Co., Ltd., TKK5401) once with the dominant hand in the standing position with maximum effort. Walking speed was measured five times, and the average value was calculated. The measurement section was 2.4 m, and a 2 m reserve track was provided in the front and back. The walking speed (m/s) was calculated by measuring the required time using a stopwatch. In addition, the instruction for normal walking was “Please walk at your usual speed.” Body composition was measured using a body composition analyzer (InBody-270, INBODY) using the bioimpedance method.

Depression was evaluated using the GDS 15 [28]. The GDS 15 consists of 15 items and is a test with two choices of “yes” or “no.” Each item is scored 1 point, and 15 points are calculated. The higher the score, the more severe the depression. According to the GDS classification, individuals with 0 to 4 points were healthy, those with 5 to 9 points had depressive symptoms, and those with 10 points or more had depression.

Statistical examination was performed using SPSS statistics version 28.0 (IBM Software Group Chicago IL). Using the 2018 and 2019 data as a baseline, the 2020 mail survey was treated as the second survey and the 2021 mail survey as the third survey. Normality was checked using the Shapiro–Wilk test and homoscedasticity was assessed using Levene’s test. The GDS 15 score at baseline was divided into two groups: the non-DS group and the DS group. The non-DS and DS groups were compared using a two-way analysis of variance. A Mann–Whitney U test was performed to evaluate the cross-group differences between the two groups at baseline, the second time, and the third time. In addition, a two-group comparison of changes in the degree of GLFS 25 was performed using the χ2 test. Furthermore, to test whether the baseline GDS score influenced the GLFS25 score in the third survey, multiple regression analysis was performed with the third GLFS-25 as the objective variable, the GDS15 score as the explanatory variable, and age and sex as confounding factors to calculate unstandardized coefficients and 95% confidence intervals Unstandardized coefficients and 95% confidence intervals were calculated. The significance level was set at *p* < 5.

## Results

Of the 504 participants, 194 returned the questionnaire (response rate, 38.5%). Of these, 187 individuals were analyzed, excluding 7 participants with a GDS score of 10 or higher. Table 1 shows the participant characteristics, such as age, sex, and physical function at baseline. There were 49 individuals (26.2%) in the DS group and 138 (73.8%) in the non-DS group. In addition, in the two-group comparison between the healthy and depressed groups at baseline, there was a significant difference only in the number of medications taken and the GDS 15 score.

**Table 1:** Participant characteristics

Items	All				DS group			Non-DS group			*p*—Value
	*n* = 187			Min. max.range	*n* = 49			*n* = 138			
Age (years)	75.5	±	5.5	[65–97]	75.4	±	5.8	75.6	±	5.5	0.806
Sex(Female(%))	144	(77.0)		-	38	(77.6)		106	(76.8)		0.543
Height (cm)	154.1	±	8.1	[140.0–178.0]	155.2	±	7.2	153.7	±	8.3	0.749
Weight(kg)	53.6	±	9.2	[35.4–83.0]	54.3	±	11.1	53.4	±	8.6	0.441
GLFS 25 (point)	7.9	±	6.7	[0–47]	8.7	±	7.9	7.6	±	6.1	0.345
SMI (kg/m2)	6.06	±	0.93	[4.2–8.3]	6.08	±	0.99	6.05	±	0.92	0.867
Grip strength (kg)	23.8	±	6.7	[10.0–50.0]	23.6	±	7.0	23.8	±	6.6	0.821
Gait speed (m/s)	1.30	±	0.25	[0.83–1.82]	1.31	±	0.20	1.30	±	0.19	0.625
Number of medications	2.5	±	2.5	[0–12]	3.1	±	2.4	2.2	±	2.40	0.034
GDS (point)	3.0	±	2.3	[0–9]	6.3	±	1.3	1.9	±	1.30	0.000
MMSE (point)	28.8	±	1.6	[23-30]	28.5	±	1.8	28.9	±	1.5	0.157

Numbers are represented as median ± standard deviation or n(%) *DS* Depressive symptoms, *GLFS 25* locomotive syndrome 25, *SMI* Skeletal muscle mass index, *GDS* Geriatric Depression Scale, *MMSE* Mini Mental State Examination

Table 2 shows the results of the two-way analysis of the variance of the longitudinal change in the total score of GLFS 25, which was the main survey item of this study.

**Table 2:** Pre, 2nd, and 3rd LS 25 score in the two groups

											**Two-way ANOVA**				Comparison of two groups for each survey period*		
											**Time effect**		**Time × group interaction**				
Item		Pre			2nd			3rd			F-value	*p*-value	F-value	*p*-value	*p*-value	*p*-value	*p*-value
GLFS-25 score	non-DS group (*n *= 138)	**7.6**	** ± **	**6.2**	**10.0**	±	**8.5**	**10.8**	** ± **	**10.5**	**22.53**	**0.000**	**2.37**	**0.095**	**0.239**	**0.027**	**0.001**
	DS group (*n* = 49)	**8.7**	** ± **	**7.9**	**13.7**	±	**10.5**	**14.9**	** ± **	**10.1**							

Numbers are median ± standard deviation　*Mann–Whitney U test

Numbers are represented as median ± standard deviation

*DS* Depressive symptoms, *GLFS* Locomotive syndrome, *ANOVA* Analysis of variance

Although no significant difference was observed between the two groups at baseline, the non-DS group scored 10.0 ± 8.5 points in the second survey, while the DS group scored 13.7 ± 10.5 points. In the third survey, the non-DS group scored 10.8 ± 10.5 points, whereas the DS group scored 14.9 ± 10.1 points. A main effect of time was observed in both groups. Regarding the difference between the two groups at each time point, the GLFS 25 score of the DS group significantly worsened in the second and third surveys.

Table 3 shows the changes in the GLFS category. Compared with the baseline, the number and percentage of those classified into the high GLFS category in both groups tended to increase in the second and third surveys. In the third survey, the DS group showed a significant decrease in the number of those classified as robust, 9 (18.4%).

**Table 3:** Two-group comparison of GLFS category changes

	Pre				2nd				3rd				Pre*^1^	2nd*^2^	3rd*^3^
	non-DS group (*n* = 138)		DS group (*n* = 49)		non-DS group (*n* = 138)		DS group (*n* = 49)		non-DS group (*n* = 138)		DS group (*n* = 49)		*p*-value	*p*-value	*p*-value
GLFS category															
Rubust	48	(34.8)	8	(16.3)	52	(37.7)	15	(30.6)	63	(33.7) **	9	(18.4) **	n.s	n.s	*p* < 0.05
GLFS1	75	(54.3)	31	(63.3)	58	(42.0)	19	(38.8)	41	(29.7)	21	(42.9)			
GLFS2	11	(8.0)	8	(16.3)	16	(11.6)	5	(10.2)	20	(14.5)	12	(24.5)			
GLFS3	4	(2.9)	2	(4.1)	12	(8.7)	10	(20.4)	14	(10.1)	7	(14.3)			

Numbers are median ± standard deviation or n (%)

*DS* Depressive symptoms, *n. s.* Not significant, *GLFS* Locomotive syndrome

^*^1–3: Statistical significance set at *p* < 0.05 using chi-square test for categorical data between 2 groups in each phase

^**^Significant difference with adjusted residual test

Table 4 shows the multiple regression analysis with baseline depression as the objective variable, GLFS-25 score in the third survey as the explanatory variable, and age and sex as confounding factors showed that baseline depression had a significant negative effect on the locomotive score in the third survey (β = 0.038 95% Confidence interval 0. 008–0.069).

**Table 4:** Force entry method multiple linear regression analysis for depressive symptoms

	β		95% Confidence Interval			*p*-value
GLFS-25	0.04	2.45	0.01	-	0.07	0.015
Age	-0.03	-1.04	-0.09	-	0.03	0.299
Sex	0.23	0.60	-0.53	-	1.00	0.548

## Discussion

This study was conducted to longitudinally investigate whether DS before the declaration of a state of emergency affected GLFS scores. The DS and non-DS groups at baseline showed a significant increase in GLFS 25 scores after the first emergency declaration. It was suggested that depression affects future increases in GLFS 25 score. Regarding the relationship between GLFS and depression, cross-sectional surveys have reported that depression is an independent factor in those with GLFS [32]. This study suggests a novel longitudinal causal relationship between GLFS 25 score and DS.

In our previous research on changes in GLFS 25 scores, we confirmed that factors related to social participation increased scores [33]. In particular, the score deteriorates because of the inability to socialize with friends and participate in local events, and it is presumed that people with depression need generous support.

Kobayashi et al., [23] reported that GLFS and comorbidities such as cerebrovascular disease, cardiovascular disease, pulmonary disease, and renal disease are risk factors. Based on our survey, common comorbidities and depression were found to be risk factors for the aggravation of GLFS. The results show the importance of quantitative evaluation of not only physical and cognitive functions but also mental health when evaluating the risks in older people. Table 2 shows that the DS group had significantly higher GLFS-25 scores than the non-DS group in the second and third surveys and among the GLFS25 questions. In a previous study, increased scores were associated with social participation-related items such as whether they were able to see friends and participate in community events[33], suggesting that during a pandemic, peacetime. The results suggest that those with poor mental health and depression may strongly refrain from communicating with others and going out.

As shown in Table 3, the percentage of those classified as having a GLFS3 increased threefold in the non-DS and DS groups compared with before and after the third survey, and individuals were classified as having a GLFS2. Compared with the pre-treatment, the proportion of those in the non-DS group increased approximately twice, and those in the DS group increased threefold during the third survey. Both groups showed an increase in the GLFS 25 score during the pandemic compared with normal times, but the score in the DS group increased by 157.4% from baseline in the second survey and increased by 171.3% in the third survey, compared with the non-DS group. There was a significant difference in score change from baseline, with a 131.6% increase in the second survey and a 142.1% increase in the third survey. Therefore, it is presumed that decreasing depression at any time may suppress the increase in GLFS 25 score. Exercise is known to be effective in reducing depression, but in recent years, it has also been found that depression can be alleviated by connecting remotely using digital technology and interacting with friends and neighbors [34].

Since there was no difference between the two groups in the GLFS 25 score and classification at baseline, it is speculated that participation in the community is likely to decrease significantly in the DS group under the state of emergency declaration. DS causes a decrease in the frequency of going out and spontaneity, making it difficult to actively invite friends or participate in events. Therefore, there is a strong need to extend support in the community to individuals in the DS group. Being unable to go out raises concerns about a decrease in physical activity [35] and deterioration of physical frailty in the future [36], and weakened connections with others may increase the risk of developing dementia [37]. In addition, severe GLFS is associated with cognitive decline [38]. Therefore, considering the new lifestyle, effective measures against infectious diseases, such as hand disinfection and wearing a mask [39, 40], should be taken sufficiently, and social activities should be resumed by older people, which will improve GLFS and cognitive function. This is an important social issue from the viewpoint of the maintenance and prevention of frailty.

This study had some limitations. First, since the pre-survey period was from August 2018 to September 2019, there were variations in the period before and after the comparison. Second, because the survey was conducted in Kaizuka City, Osaka Prefecture, further surveys are required to generalize the results. Moreover, the response rate was only 38.5% because the survey was conducted by mail. Third, the responses might be biased because the survey was self-administered. Fourth, it is necessary to consider that 310 (61.5%) of the 504 who did not reply did not participate in the mail survey. However, we have also confirmed that the motor function, cognitive function, and depression of the group that did not participate in the mail survey were not significantly different from those of the group that did participate in the mail survey at baseline; we believe that these data can be treated as generalized data for the older adults living in the community. It is necessary to carefully observe the changes in mental health by conducting additional surveys. The results of this study indicate that professionals involved in promoting the health of older adults living in the community need to carefully assess their mental health as well as their motor and cognitive functions during normal times, and at the same time, should be aware that in an emergency situation such as a pandemic, those with depressive tendencies are likely to be strongly affected.

## Conclusions

Our results revealed that the GLFS 25 score significantly increased in community-dwelling older adults under the declaration of a state of emergency. In particular, those with depression showed significantly high scores. Evaluating depressed people from normal times and providing substantial social support may effectively reduce the deterioration of the GLFS 25 score and motor dysfunction.

## Data Availability

The datasets used and/or analyzed during the current study are available from the corresponding author on reasonable request.

## Abbreviations

COVID-19: Coronavirus disease 2019
GLFS: Locomotive syndrome
DS: Depressive symptoms
GDS: Geriatric Depression Scale
